# Redroot Pigweed (*Amaranthus retroflexus* L.) and Lamb’s Quarters (*Chenopodium album* L.) Populations Exhibit a High Degree of Morphological and Biochemical Diversity

**DOI:** 10.3389/fpls.2021.593037

**Published:** 2021-01-29

**Authors:** Shiva Hamidzadeh Moghadam, Mohammad Taghi Alebrahim, Ahmad Tobeh, Mehdi Mohebodini, Danièle Werck-Reichhart, Dana R. MacGregor, Te Ming Tseng

**Affiliations:** ^1^Department of Agronomy and Plant Breeding, Faculty of Agriculture and Natural Resources, University of Mohaghegh Ardabili, Ardabil, Iran; ^2^Department of Horticultural Sciences, Faculty of Agriculture and Natural Resources, University of Mohaghegh Ardabili, Ardabil, Iran; ^3^Institute of Plant Molecular Biology, CNRS, University of Strasbourg, Strasbourg, France; ^4^Department of Biointeractions and Crop Protection, Rothamsted Research, Harpenden, United Kingdom; ^5^Department of Plant and Soil Sciences, Mississippi State University, Starkville, MS, United States

**Keywords:** cluster analysis, climate change, morphological and biochemical traits, noxious weeds, principal component analysis

## Abstract

*Amaranthus retroflexus* L. and *Chenopodium album* L. are noxious weeds that have a cosmopolitan distribution. These species successfully invade and are adapted to a wide variety of diverse climates. In this paper, we evaluated the morphology and biochemistry of 16 populations of *A. retroflexus* L. and 17 populations of *C. album* L. Seeds from populations collected from Spain, France, and Iran were grown together at the experimental field of the agriculture research of University of Mohaghegh Ardabili, and a suite of morphological traits and biochemical traits were assessed. Among the populations of *A. retroflexus* L. and of *C. album* L. were observed significant differences for all the measured traits. The number of branches (BN) for *A. retroflexus* L. (12.22) and inflorescence length (FL; 14.34) for *C. album* L. were the two characteristics that exhibited the maximum coefficient of variation. Principal component analysis of these data identified four principal components for each species that explained 83.54 (*A. retroflexus* L.) and 88.98 (*C. album* L.) of the total variation. A dendrogram based on unweighted neighbor-joining method clustered all the *A. retroflexus* L. and *C. album* L. into two main clusters and four sub-clusters. Canonical correlation analysis (CCA) was used to evaluate relationships between climate classification of origin and traits. Similarly, the measured characteristics did not group along Köppen climate classification. Both analyses support the conclusion that *A. retroflexus* L. and *C. album* L. exhibit high levels of diversity despite similar environmental histories. Both species also exhibit a high diversity of the measured biochemical compounds indicating that they exhibit different metabolic profiles even when grown concurrently and sympatrically. Several of the biochemical constituents identified in our study could serve as effective indices for indirect selection of stresses resistance/tolerance of *A. retroflexus* L. and *C. album* L. The diversity of the morphological and biochemical traits observed among these populations illustrates how the unique selection pressures faced by each population can alter the biology of these plants. This understanding provides new insights to how these invasive plant species successfully colonize diverse ecosystems and suggests methods for their management under novel and changing environmental conditions.

## Introduction

*Amaranthus retroflexus* L. (redroot pigweed) and *Chenopodium album* L. (lamb’s quarters) are fast-growing weedy annual plants that belong to the *Amaranthaceae* family. They are both listed among the most common dicotyledonous weeds in the world and are widely distributed in many agricultural areas (Horak and Loughin, 2000; Alebrahim et al., 2012) where they cause significant problems. They severely reduce the yield of the crops in which they grow while their destructive growth and allelopathic activity make them very competitive resulting in significant decreases in crop yield and quality (Ma et al., 2015; Bajwa et al., 2019).

*Amaranthus retroflexus* is a C_4_ plant (Baskin and Baskin, 1978) considered to be native to North America, but it now is distributed worldwide (Frankton and Mulligan, 1987). Where it has been introduced, this annual weed is a casual weed on cultivated land and in waste places such as rubbish tips (Clapham et al., 1987; Stace, 1997; Bond et al., 2007). It grows best at higher temperatures, light intensities, and nitrogen levels (Costea et al., 2003). *A. retroflexus* has a negative influence on row crops, such as sugar beet (Brimhall et al., 1967), soybean (Dieleman et al., 1995), potato (Weaver, 1991; Azadbakht et al., 2017), cotton (Buchanan et al., 1980), and corn (Kenz̆ević et al., 1995).

*Chenopodium album* is native to Western Asia (Poonia and Upadhayay, 2015) but even in the early 1950s was considered to be one of the five most widely distributed plants in the world (Williams, 1963). *C. album* is a weed in crops including wheat, barley, mustard, and gram (Sarabi et al., 2013; Jabran et al., 2017). This weed is low growing while the cultivated plants in which it grows are frequently tall and leafy (Bhattacharjee, 2001).

Both species interfere with human land use as they are successful colonizers and have considerable impact on plant growth (Garbari and Pedulla, 2001). They are adapted to highly unstable and unpredictable environments, can compete with other plants for nutrients, water, light, and space through different survival tactics, and can harbor crop pests or diseases (Rodenburg et al., 2010). The number of herbicides that can be used to control them is limited and these herbicides are not very efficient (Alebrahim et al., 2011). Quantifying how much morphological and biochemical diversity is exhibited in populations from different geographical locations is necessary to design and employ effective management practices (Jannatabadi et al., 2014). In particular, it is still unclear if the performance of invasive species is driven by ecological processes, evolutionary processes, or both (Pearson et al., 2018).

The ability of plants to vary their morphological traits has long been recognized as a beneficial survival strategy that enables plants to acclimatize to changing habitats (Gambino and Vilela, 2011). Plants exhibit a high degree of phenotypic plasticity which enables them to incorporate information from the environment into decisions about their morphology. Changes in morphology are often connected to the conditions under which the plant is growing (Mandák et al., 2011). For instance, root (MacGregor et al., 2008) and shoot (Teichmann and Muhr, 2015) architecture can vary dramatically between isogenic plants in response to different environmental conditions. Hence, the same species of plant can occupy and be maintained in diverse habitats by appropriately adjusting plant morphology (Urbas and Zobel, 2000).

That said, plants are genetically constrained in the forms that theycan adopt; otherwise, taxonomic classification of plants would beimpossible. An understanding of a plant’s morphological andbiochemical variability is useful for designing management andconservation strategies that balance endemic with invasive species asit explains colonization history through genetic diversity andpopulation structures(Thompson, 1999).

In this study, we aimed to better understand the colonization history and capacity for invasiveness of *A. retroflexus* and *C. album* by characterizing a suite of morphological and biochemical traits in populations of collected from contrasting habitats. We choose traits that are associated with successful invasions; for instance, specific leaf area (SLA) is a key functional trait representing the amount of light-capturing surface area and thus is used widely to estimate plant carbon acquisition efficiency provides a useful framework to assess invasive plant responses to climate change and the population’s variability (Colautti and Barrett, 2013). This collection was examined for morphological and biochemical variations in order to understand the strategies that have enabled their successful invasion into a wide range of habitats by providing a selective advantage for competitiveness of these varied environments. We hypothesized that (1) populations of *A. retroflexus* L. and *C. album* L. from different invaded seed source regions would exhibit variation in plant traits when grown in common garden and (2) populations grown from seeds of the same type of climate zone would display characters more similar to those from the different climate zones. We have found that the biochemical compounds and morphological traits vary significantly in both *A. retroflexus* and *C. album* even when grown concurrently and sympatrically and that the population’s original climate could not accurately predict its morphology or biochemistry. Although variability among populations is expected, these species are able to grow in a wide range of environmental conditions. This knowledge indicates that a “Universal Management Regime” will not be suitable for these species.

## Materials and Methods

### Plant Materials

**FIGURE 1:**
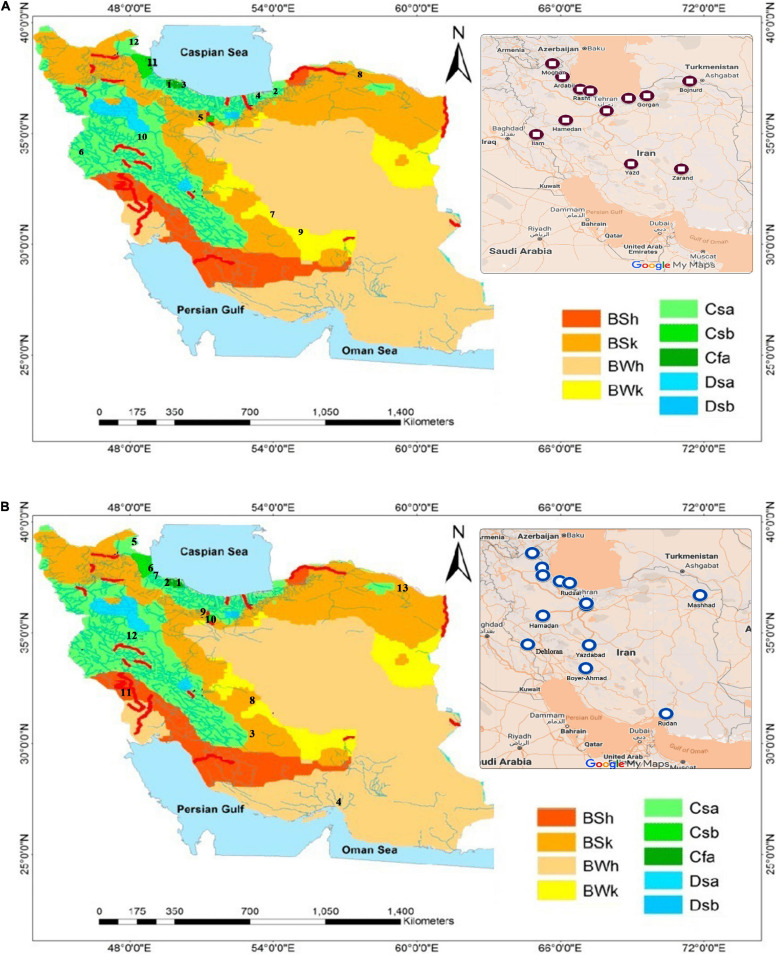
**(A)** Sampling sites of *A. retroflexus* populations according to the Koppen–Geiger classification (1990–2014) (Raziei, 2017). Numbers on map are population names based on Table 1. **(B)** Sampling sites of *C. album* populations according to the Koppen–Geiger classification (1990–2014) (Raziei, 2017). Numbers on map are population names based on Table 1.

In order to investigate the morphological and biochemical characteristics of these weeds, seeds of 16 *A. retroflexus* and 17 *C. album* populations were collected in 2016 and 2017 from different provinces of Iran, Spain, and France (Table 1). The seeds provided by Research Institute of Forests and Rangelands (RIFR) and UMR Agroecology (INRA Dijon) were cultivated at the experimental field of the agriculture research of University of Mohaghegh Ardabili (38°19′N 48°20′E) (Figures 1A,B).

**TABLE 1:** Region name, country of origin, geographical coordinates, and Köppen climate classification of *A. retroflexus* and *C. album* populations used herein.

***A. retroflexus***				
**No.**	**Region name**	**Origin**	**Coordinate**	**Köppen climate classification**

1	Rasht	Iran	37°16′05 N 49°35′20 E	Humid subtropical climate (Cfa)
2	Gorgan	Iran	36°45′06 N 54°21′40 E	Hot summer mediterranean climate (Csa)
3	Rudsar	Iran	37°08′16 N 50°17′10 E	Humid subtropical climate (Cfa)
4	Sari	Iran	36°33′57 N 53°03′31 E	Hot summer mediterranean climate (Csa)
5	Shahr-e-Rey	Iran	35°34′37 N 51°27′44 E	Cold semi-arid climate (Bsk)
6	Ilam	Iran	33°38′05N 46°24′54 E	Hot summer mediterranean climate (Csa)
7	Yazd	Iran	31°10′97 N 53°11′97 E	Cold desert climate (Bwk)
8	Bojnurd	Iran	37°53′74 N 57°24′96 E	Cold semi-arid climate (Bsk)
9	Zarand	Iran	30°47′27 N 56°50′10 E	Cold desert climate (Bwk)
10	Hamedan	Iran	34°47′50 N 48°30′45 E	Hot summer mediterranean climate (Csa)
11	Ardabil	Iran	38°14′54 N 48°17′03 E	Hot-summer humid continental climate (Dsa)
12	Moghan	Iran	39°13′00 N 47°33′53 E	Humid subtropical climate (Cfa)
13	France	France	47°19′20 N 5°2′28 E	Humid subtropical climate (Cfa)
14	Spain 1	Spain	37°53′18 N 4°46′38 W	Hot summer mediterranean climate (Csa)
15	Spain 2	Spain	37° 53′15 N 4° 46′35 W	Hot summer mediterranean climate (Csa)
16	Spain 3	Spain	37° 53′14 N 4° 46′45 W	Hot summer mediterranean climate (Csa)
*C. album*				
1	Rudsar	Iran	37°08′13 N 50°16′52 E	Humid subtropical climate (Cfa)
2	Rasht	Iran	37°16′03 N 49°35′08 E	Humid subtropical climate (Cfa)
3	Boyer-Ahmad	Iran	30°53′47 N 51°24′96 E	Hot semi-arid climate (Bsh)
4	Rudan	Iran	27°25′44 N 57°10′45 E	Hot desert climate (Bwh)
5	Moghan	Iran	39°12′03 N 47°34′24 E	Humid subtropical climate (Cfa)
6	Kivi	Iran	37′41′02 N 48°20′53 E	Hot summer mediterranean climate (Csa)
7	Ardabil	Iran	38°12′44 N 48°17′38 E	Hot-summer humid continental climate (Dsa)
8	Yazdabad	Iran	32°39′41 N 51°41′21 E	Cold semi-arid climate (Bsk)
9	Shahr-e-Ray	Iran	35°34′22 N 51°27′ 44 E	Cold semi-arid climate (Bsk)
10	Tehran	Iran	35°41′13 N 51°26′22 E	Cold semi-arid climate (Bsk)
11	Dehloran	Iran	32°41′49 N 47°16′05 E	Hot semi-arid climate (Bsh)
12	Hamadan	Iran	34°49′46 N 48°19′ 47 E	Hot summer mediterranean climate (Csa)
13	Mashhad	Iran	36°16′24 N 59°38′16 E	Cold semi-arid climate (Bsk)
14	Spain 1	Spain	37°53′ 15 N 4°46′35 W	Hot summer mediterranean climate (Csa)
15	Spain 2	Spain	37°53′ 14 N 4°46′45 W	Hot summer mediterranean climate (Csa)
16	France 1617	France	47°19′20 N 5°2′28 E	Humid subtropical climate (Cfa)
17	France 1499	France	47°19′29 N 5°2′22 E	Humid subtropical climate (Cfa)

To assess the morphological and biochemical traits, seeds from the each population were germinated in plastic trays containing a growing medium without fertilizers. Three weeks after sowing, five plants per population were selected and planted outdoors at the experimental field of the agriculture research of University of Mohaghegh Ardabili during the summer of 2018. Three replicates plots with five seedlings per replicate were planted in each plot. Seeds were planted at a distance of 20 cm in row and 30 cm between rows. At the end of the growing season, 12 morphological traits were evaluated on three randomly selected plants: plant height (PH), inflorescence length (FL), leaf length (LL), leaf width (LW), leaf area (LA), number of leaves (LN) number of branches (BN), diameter of stem (SD), fresh weight (FW), dry weight (DW), SLA, and seed weight (SW). For the analyses of some of the biochemical parameters: chlorophyll a (Ca), chlorophyll b (Cb), total chlorophyll (TC), carotenoid (Car) and total protein content (TP), catalase (CAT), peroxidase (POD), and polyphenol oxidase (PPO); the fresh leaf samples were collected and stored at −70°C until analyses.

### Determination of Specific Leaf Area

Samples were randomly selected from each plant. The surface area of each leaf [S (cm^2^)] was measured. Then, the leaf was dried (70°C, 48 h) for dry mass measurements [M (g)]. The surface area (S) was divided by the mass (M) to obtain the SLA.

### Determination of Leaf Photosynthetic Pigments

To determine leaf photosynthetic pigment content, approximately 0.25 g of fresh plant leaf sample was homogenized in 5 ml 80% acetone. Homogenates were centrifuged at 10,000 r/min for 15 min at 4°C and 0.25 ml of the clarified supernatant was mixed with 2.5 ml of 80% acetone. The absorbance of acetone extracts was measured at 662, 645, and 470 nm for determination of Ca, Cb, and Car content using a spectrophotometer. The leaf photosynthetic pigments were expressed as mg g^–1^ on FW basis using the formula listed below (Lichtenthaler and Wellburn, 1983).

$$\begin{array}{c} C\,a=12.25\,A-2.798\,A\,646.8\\ C\,b=21.50\,A\,646.8-5.10\,A\,663.2\\ T\,C=C\,a+C\,b\\ C\,a\,r=\left(1000\,A\,470-1.82\,C\,a-85.02\,C\,b\right)/198 \end{array}$$

### Determination of Protein Content

Total protein content was measured using the method of Bradford (1976) using bovine serum albumin (BSA) standard as a standard. Protein concentrations were measured using a NanoDrop spectrophotometer (Thermo One C., Termo scientific, Inc., United States) at 595 nm.

### Extraction of Antioxidant Enzymes

To extract proteins for antioxidant enzyme analysis, 200 mg of leaf samples was flash-frozen in liquid nitrogen and homogenized in 10 ml of Tris-HCl buffer (pH 7.5, 0.1 M). The homogenate was centrifuged at 13,000 r/min for 15 min at 4°C and supernatants collected to determine CAT, POD, and PPO activities using established protocols described in Sudhakar et al. (2001).

### Determination of Enzymatic Activities

To determine CAT activity (EC 1.11.1.6), the method described by Chance and Maehly (1995) was used with the following modifications. Degradation of H_2_O_2_ in a reaction medium containing 300 μM tris buffer (pH 7.5), 100 μM H_2_O_2_ and 1 ml of plant extract mixed in an ice bath was monitored at 240 nm for 2 min. The same reaction medium free of plant extract was used as a blank.

The activity of PPO (EC 1.10.3.1) was determined according to Kar and Mishra (1976) with minor modifications. The reaction medium consisted of the same assay mixture as that of POD without H_2_O_2_ and was incubated at 25°C. Readings were taken at 420. Enzymatic activities were expressed in absorbency units (unit mg^–1^ protein min^–1^).

The activity of POD (EC 1.11.1.7) was determined by reading absorbance at 420 nm according to Kar and Mishra (1976) with minor modifications. The reaction mixture consisted of 125 μM tris buffer (pH 7.5), 50 μM pyrogallol, and 50 μM H_2_O_2_, and 1 ml of the total plant extract was incubated for 5 min at 25°C. As a control, the same reaction medium was incubated in the absence of plant extract under the same conditions.

### Statistical Analysis

ANOVA tests were performed for each morphological and biochemical parameter using SAS package (9.3 SAS Institute, Inc., United States). The simple correlation coefficient among the studied variables using the Pearson’s correlation coefficient method, principal component analysis, and scatter plot of loadings corresponding to the first three principal components were made using the SPSS software (22, SPSS, Inc., Chicago, IL, United States). Unweighted pair-group method of arithmetic averages (UPGMA) method was performed using SPSS 16 to determine the individual relationship among populations by adopting the Ward method based on squared Euclidean distance and to determine the best cut-off point of the dendrogram, a canonical discriminant function analysis (Manly, 2005). Canonical correlation analysis (CCA) was used to evaluate relationships between Köppen climate classification (Raziei, 2017) and morphological and biochemical traits by PROC CANCORR procedure of SAS program version 9.3.

## Results

### Morphological Traits

To determine if the populations of *A. retroflexus* and *C. album* exhibited different morphological traits, PH, FL, LL, LW, LA, LN, BN, SD, FW, DW, SLA, and SW were measured. All of the measured morphological traits differed significantly among the populations of *A. retroflexus* and *C. album* (Tables 2A,B).

**TABLE 2:** Variance analysis of the evaluated traits in *A. retroflexus* **(A)** and *C. album* **(B)** populations.

**(A)**													

**Source of variation**	**Degrees of freedom**	**Mean squares**											
		
		**PH**	**FL**	**LL**	**LW**	**LA**	**LN**	**BN**	**SD**	**FW**	**DW**	**SLA**	**SW**
Replication	2	3^ns^	0.41^ns^	1.38^ns^	0.36**	33.17**	159.5**	1.75^ns^	0.36**	31.4^ns^	0.94^ns^	0.77^ns^	0.001^ns^
Population	15	1302.4**	163.2**	21.1**	4.4**	786.62**	1470.61**	10.3**	18.07**	1455.67**	50.16**	94.6**	0.57**
Error	30	5.68	0.696	0.3	0.05	5.18	66.25	0.77	0.33	41.42	1.42	2.09	0.005
CV		4.8	8.3	9.84	8.06	11.66	11.83	12.22	10.26	11.99	12.09	11.9	7.1

**Source of variation**	**Degrees of freedom**	**Mean squares**							
		
		**Ca**	**Cb**	**TC**	**Car**	**TP**	**CAT**	**POD**	**PPO**

Replication	2	0.003^ns^	0.0008^ns^	0.003^ns^	0.0002^ns^	0.0016^ns^	0.0039**	0.001^ns^	0.0007^ns^
Population	15	4.21**	1.86**	7.38**	0.4**	0.4**	0.17**	0.044**	0.018**
Error	30	0.01	0.0004	0.011	0.0002	0.004	0.0006	0.001	0.0004
CV		2.87	1.32	1.95	1.21	8.73	1.84	3.28	1.29

**(B)**													

**Source of variation**	**Degrees of freedom**	**Mean squares**											
		
		**PH**	**FL**	**LL**	**LW**	**LA**	**LN**	**BN**	**SD**	**FW**	**DW**	**SLA**	**SW**

Replication	2	74.43**	5.34*	0.05^ns^	0.04^ns^	2.12^ns^	142.82^ns^	1.11^ns^	0.011^ ns^	13.47^ns^	0.03^ns^	0.47^ns^	0.002^ns^
Population	16	1567.97**	47.8**	6.55**	4.46**	209.75**	4829.2**	30.34**	11.03**	5735.64**	169.8**	193.3**	2.09**
Error	32	6.16	1.58	0.06	0.019	0.98	56.55	0.47	0.13	28.46	0.87	25.96	0.006
CV		4.26	14.34	6.56	7.99	12.73	10.88	7.58	7.21	10.44	10.58	7.6	8.29

**Source of variation**	**Degrees of freedom**	**Mean squares**							
		
		**Ca**	**Cb**	**TC**	**Car**	**TP**	**CAT**	**POD**	**PPO**

Replication	2	0.0002^ns^	0.0017^ns^	0.0017^ns^	0.003*	0.00007^ns^	0.001^ns^	0.004^ns^	0.0002^ns^
Population	16	4.58**	1.26**	11.23**	0.71**	0.43**	0.26**	0.047**	0.19**
Error	32	0.0006	0.0005	0.001	0.0008	0.0013	0.0006	0.0003	0.0002
CV		0.85	1.88	0.78	2.27	7.41	2.41	2.11	0.97

*ns: not significant, ^∗^significant at *p* < 0.05, ^∗∗^significant at *p* < 0.01. PH, plant height; FL, inflorescence length; LL, leaf length; LW, leaf width; LA, leaf area; LN, number of leaves; BN, number of branches; SD, diameter of stem; FW, fresh weight; DW, dry weight; SW, seed weight; Ca, chlorophyll a; Cb, chlorophyll b; TC, total chlorophyll; Car, carotenoid content; TP, total protein content; CAT, catalase activity; POD, peroxidase activity; PPO, polyphenol oxidase.*

#### A. retroflexus

**FIGURE 2:**
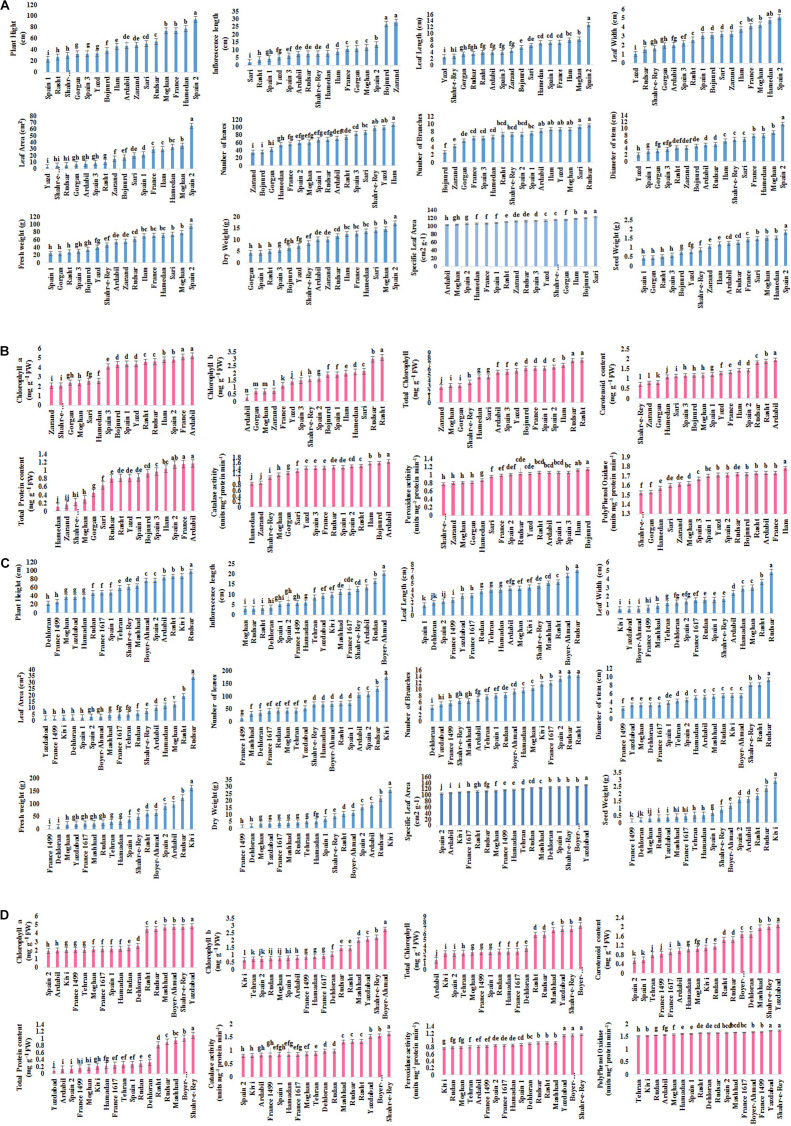
**(A)** Frequency distribution for each morphological trait in *A. retroflexus* populations. **(B)** Frequency distribution for each biochemical trait in *A. retroflexus* populations. **(C)** Frequency distribution for each morphological trait in *C. album* populations. **(D)** Frequency distribution for each biochemical trait in *C. album* populations.

Mean comparison of populations indicated shortest PH (22.6 cm) in Spain 1 and longest (93.6 cm) in Spain 2. Zarand showed the maximum FL (28 cm), followed by Bojnurd (26.63 cm), while minimum (1.96 cm) was noted in Sari. The LL, LW, and LA were highest (12.77 cm, 5.1 cm, and 65.08 cm^2^, respectively) in Spain 2 and lowest (2.5 cm, 1 cm, and 2.5 cm^2^, respectively) in Yazd. The least numbers of leaves and branches (34.66 and 2.67, respectively) were obtained in Zarand and Bojnurd, and the highest number of leaves and branches (107 and 9.67, respectively) in Ilam and Rudsar. The thickest shoot (11.32 cm) was measured in Spain 2 and thinnest (1.99 cm) in Yazd. Spain 2 showed the highest FW and DW (95.36 and 17.17 g, respectively) while the lowest (24.15 and 4.29 g, respectively) was found for FW and DW in Gorgan. The highest SLA recorded in Sari (122.35 cm^2^ g^–1^) followed by Bojnurd (120.24 cm^2^ g^–1^) and lowest (103.19 cm^2^ g^–1^) in Ardail. SW was the highest (1.83 g) in Spain 2 and the lowest in Spain 1 (0.43 g), followed by Gorgan (0.42 g) (Figure 2A).

#### C. album

Mean comparison of populations showed minimum PH in Dehloran (22 cm) and maximum in Rudsar (97.5 cm). Maximum FL was observed in Boyer-Ahmad (20.4 cm) and minimum (3.1 cm) was noted for Moghan, followed by Rudsar (3.2 cm) and Rasht (3.3 cm). The shortest LL (1.6 cm) was observed for Spain 2 (1.6 cm) followed by Dehloran (2 cm), and the longest for Rudsar (7.1 cm). The widest leaves were (4.83 cm) in Rudsar, and narrowest (0.5 cm) in Kivi, Yazdabad, and Boyer-Ahmad. Rudsar showed the maximum LA (34.33 cm^2^), while minimum (1.63 cm^2^) was noted in Yazdabad, followed by France 1499 (1.65 cm^2^), Kivi (2.18 cm^2^), Dehloran, and Spain 2 (2.5 cm^2^). Largest LN and BN (175 and 14.33, respectively) were recorded for Kivi, Rudsar, and Rasht, and smallest number (14.66 and 4.33, respectively) was observed in Dehloran. The thickest shoot (9.23 cm) was in Rudsar and thinnest (2.48 cm) in France 1499. Kivi showed the highest FW and DW (161.07 and 27.72 g, respectively) and France 1499 the lowest (3.74 and 0.64 g, respectively), followed by Dehloran (8.53 and 1.49 g, respectively). Yazdaad had the highest SLA (133.33 cm^2^ g^–1^), while lowest (104.09 cm^2^ g^–1^) was recorded in Spain 2. The Kivi showed the highest SW (2.91 g) and the lowest (0.076 g) was observed for France 1499, followed by Dehloran (0.16 g) (Figure 2C).

### Biochemical Parameters

To determine if the populations of *A. retroflexus* and *C. album* exhibited different biochemical traits, Ca, Cb, TC, Car content, TP, CAT activity, POD activity, and PPO were measured. Like the morphological traits, these biochemical traits all differed significantly among the populations of *A. retroflexus* and *C. album*.

#### A. retroflexus

The highest Ca content (5.21 mg g^–1^ FW) was detected in Ardabil, which was equal with France (5.12 mg g^–1^ FW) and the minimum (2.06 mg g^–1^ FW) in Zarand. Rasht had the highest Cb content (3.11 mg g^–1^ FW), and the lowest (0.28 mg g^–1^ FW) was found for Ardabil. The highest TC content (7.69 mg g^–1^ FW) was recorded in Rasht, which was equal to Rudsar (7.61 mg g^–1^ FW), while it was at lowest (2.82 mg g^–1^ FW) in Zarand. The Ardabil had the highest total Car content (1.95 mg g^–1^ FW), while the lowest (0.71 mg g^–1^ FW) was in Shahr-e-Ray. The maximum total soluble protein content (1.17 mg g^–1^ FW) was recorded in Ardabil, followed by France (1.16 mg g^–1^ FW), and the lowest (0.11 mg g^–1^ FW) was recorded in Hamedan, followed by Zarand (0.16 mg g^–1^ FW). The highest CAT activity (1.65 units mg^–1^ protein min^–1^) was detected in Ardabil, and lowest (0.85 units mg^–1^ protein min^–1^) in Hamedan, followed by Kerman (0.88 units mg^–1^ protein min^–1^). The highest POD activity (1.14 units mg^–1^ protein min^–1^) was recorded in Bojnurd followed by Ilam (1.12 units mg^–1^ protein min^–1^) and the lowest (0.77 units mg^–1^ protein min^–1^) in Shahr-e-Ray followed by Zarand, Moghan (0.81 units mg^–1^ protein min^–1^), and Gorgan (0.82 units mg^–1^ protein min^–1^). The highest PPO activity (1.78 units mg^–1^ protein min^–1^) was recorded in Ilam, and the lowest (1.52 units mg^–1^ protein min^–1^) in Shahr-e-Ray followed by Gorgan (1.53 units mg^–1^ protein min^–1^) (Figure 2B).

#### C. album

The largest concentration Ca (4.79 mg g^–1^ FW) was recorded in Yazdabad and the lowest (1.98 mg g^–1^ FW) in Spain 2 followed by Ardabil (2 mg g^–1^ FW). The Boyer Ahmad had the highest Cb and TC content (2.75 and 7.46 mg g^–1^ FW, respectively), while the lowest (0.66 and 2.7 mg g^–1^ FW, respectively) was found in Kivi. The highest total Car (2.09 mg g^–1^ FW) was recorded in Yazdabad and the lowest was detected in Spain 2 (0.56 mg g^–1^ FW). The Shahr-e-Rey had the highest total soluble protein content (1.1 mg g^–1^ FW), while the lowest was found (0.08 mg g^–1^ FW) in Yazdabad. The highest CAT activity (1.64 units mg^–1^ protein min^–1^) was measured in the Shahr-e-Ray and the lowest in Spain 2 and Kivi (0.8 units mg^–1^ protein min^–1^) followed by France 1499 and Ardabil (0.83 units mg^–1^ protein min^–1^). The Boyer Ahmad, Yazd Abad, and Shahr-e-Ray had the highest (1.1 units mg^–1^ protein min^–1^) POD activity, while the lowest (0.77 units mg^–1^ protein min^–1^) was in Kivi, followed by Rudan and Moghan (0.8 units mg^–1^ protein min^–1^). The highest PPO activities (1.7 units mg^–1^ protein min^–1^) were in Shahr-e-Ray and Yazdabad, and the lowest in Kive and Tehran (1.51 units mg^–1^ protein min^–1^) (Figure 2D).

### Correlation Among Measured Traits

#### A. retroflexus

The correlations coefficients among the morphological and biochemical populations are presented in Table 3A. PH showed significant positive correlation with the LA (*r* = 0.8), SD (*r* = 0.87), FW (*r* = 0.9), and SW (*r* = 0.9). FL was significantly negatively correlated with the LN (*r* = −0.69) and BN (*r* = −0.74). LL showed significantly positively correlated with LA (*r* = 0.98), SD (*r* = 0.78), FW (*r* = 0.69), and SW (*r* = 0.63). The LA was positively correlated with SD (*r* = 0.83), FW (*r* = 0.73), and SW (*r* = 0.68). The LN was positively correlated with the BN (*r* = 0.69). SD showed highly significant positive correlated with FW (*r* = 0.87), but had negative correlation with SW (*r* = −0.85).

**TABLE 3:** Correlation matrices for the morphological and biochemical traits in *A. retroflexus* **(A)** and *C. album* **(B)**.

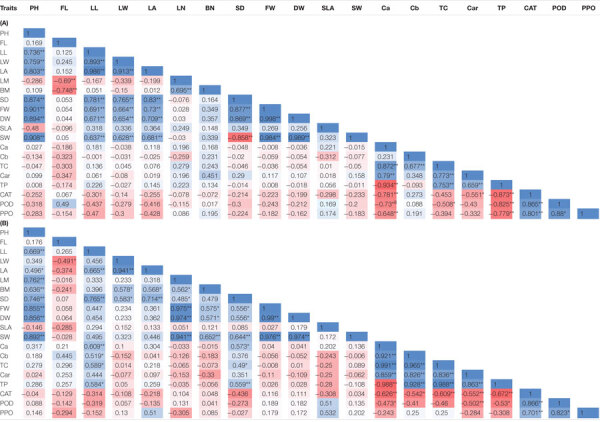

**Correlation is significant at the 0.01 level (two-tailed)

*Correlation is significant at the 0.05 level (two-tailed).

*Positive correlations are indicated in blue and negative correlations in red.*

Chlorophyll a content showed highly significant positive correlation with TC content (*r* = 0.87), Car (*r* = 0.79), total protein (*r* = 0.93), and highly significant negative correlation with CAT (*r* = −0.78), POD (*r* = −0.73), and PPO activity (*r* = −0.64). Cb content was significantly positively correlated with TC content (*r* = 0.67). Car content showed significant positive correlation with TC content (*r* = 0.65) and significant negative correlation with CAT (*r* = −0.55). TC content showed positive correlation with Car (*r* = 0.77) and total protein (*r* = 0.75), and negative correlation with POD (*r* = −0.5). Total soluble protein content was significantly negatively correlated with CAT (*r* = −0.87), POD (*r* = −0.82), and PPO (*r* = −0.77) activity. CAT activity was positively correlated with POD (*r* = 0.86) and PPO (*r* = 0.8) activity. POD activity was positively correlated with PPO (*r* = 0.88) activity (Table 3A).

#### C. album

Plant height was positively correlated with LA (*r* = 0.49), LN (*r* = 0.76), BN (*r* = 0.63), SD (0.74), FW (*r* = 0.85), and SW (*r* = 0.89). In addition, FL was significantly negatively correlated with LW (*r* = 0.49). LA was positively correlated with the BN (*r* = 0.58) and SD (0.58). The LN showed positive correlation with BN (*r* = 0.56), SD (0.48), FW (*r* = 0.97), and SW (*r* = 0.94). BN was significantly positively correlated with FW (*r* = 0.57) and SW (*r* = 0.65). SD was positively correlated with FW (*r* = 0.55), SW (*r* = 0.64), Ca content (*r* = 0.57), TC content (*r* = 0.49), and total protein (*r* = 0.55). SLA was significantly positively correlated with POD (*r* = 0.51) and PPO (*r* = 0.53) activity.

Chlorophyll a content was significantly negatively correlated with the CAT activity (*r* = −0.62) while a positive correlation with Cb content (*r* = 0.92), TC content (*r* = 0.99), Car (*r* = 0.85), and total protein (*r* = 0.9). Cb content showed negative correlation with CAT activity (*r* = −0.54), while a positive correlation with TC content (*r* = 0.96), Car (*r* = 0.82), and total protein (*r* = 0.92). Car was significantly positively correlated with total protein (*r* = 0.86), but negatively correlated with CAT (*r* = −0.55) and POD (*r* = −0.5) activity. Total soluble protein content was significantly negatively correlated with CAT (*r* = −0.67) and POD (*r* = −0.53) activity. CAT activity was positively correlated with POD (*r* = 0.86) and PPO (*r* = 0.7) activity. POD activity was positively correlated with PPO (*r* = 0.82) activity (Table 3B).

### Principal Component Analysis (PCA)

#### A. retroflexus

In this evaluation, effective traits were divided into four components accounting for 88.22% of the total observed variance. Loading values higher than 0.5 were considered significant as suggested by Wu et al. (2016). Four principal components (PC1, PC2, PC3, and PC4) explained together more than 83.54% of the total variation (Table 4A). PC1 related with PH, LL, LW, LA, SD, FW and DW, SLA, and SW explained 35.2% of the total variability. Component PC2 was associated with Ca, Cb, TC, Car, and TP and accounted for 24.17% of the total variability. Component PC3 was mainly associated with FL, LN, and BN and accounted for 14.951% of the total variability. Component PC4 showed the integration with CAT, POD, and PPO activity and explained 9.213% of the total variability. Hence, the morphological and biochemical parameters could effectively explain the existing variability.

**TABLE 4:** Eigen values, variance (%), and cumulative variance (%) for four principal components obtained from PCA and significant characters within each component in the studied *A. retroflexus* **(A)** and *C. album* **(B)**.

	**Principal component**			
	
**Characteristics**	**1**	**2**	**3**	**4**
**(A)**				
PH	**0.896**	0.266	0.166	0.099
FL	0.184	−0.31	−**0.668**	−0.19
LL	**0.602**	0.504	0.331	−0.137
LW	**0.942**	0.21	0.03	−0.009
LA	**0.855**	0.34	0.228	0.098
LN	−0.11	0.099	**0.63**	0.018
BN	0.540	−0.153	**0.63**	−0.017
SD	**0.592**	0.518	0.45	−0.148
FW	**0.978**	0.03	0.032	0.094
DW	**0.979**	−0.013	0.03	0.098
SLA	−**0.533**	0.466	−0.358	−0.523
SW	**0.96**	0.062	0.151	0.092
Ca	0.065	**0.962**	0.086	−0.179
Cb	−0.015	−0.191	−0.135	**0.945**
TC	0.039	0.008	0.973	**0.973**
Car	−0.12	**0.878**	−0.088	−0.197
TP	−0.042	**0.950**	0.032	−0.249
CAT	−0.116	**0.795**	0.048	0.487
POD	0.098	−**0.899**	−0.328	−0.08
PPO	0.143	**0.931**	0.089	0.073
Eigen variance	6.04	5.89	2.983	2.87
Percentage of variance	30.2	29.49	14.91	14.37
Cumulative percentage	30.2	59.694	74.6	88.98
**(B)**				
PH	**0.943**	0.087	−0.54	−0.131
FL	0.101	−0.077	−**0.873**	−0.145
LL	**0.842**	0.282	−0.183	0.098
LW	0.**857**	0.065	−0.332	0.111
LA	**0.885**	0.220	−0.201	0.066
LN	−0.158	0.110	**0.814**	0.104
BN	0.183	0.000	**0.933**	0.007
SD	**0.934**	0.036	0.027	−0.043
FW	**0.937**	0.010	0.190	−0.096
DW	**0.933**	−0.020	0.205	0.086
SLA	**0.427**	−0.193	−0.059	−0.372
SW	**0.924**	−0.007	0.197	−0.114
Ca	−0.052	**0.925**	0.155	0.172
Cb	−0.025	**0.946**	0.000	0.232
TC	0.052	**0.700**	0.234	0.605
Car	0.399	**0.436**	−0.307	−0.005
TP	−0.048	**0.97**	0.129	0.073
CAT	−0.246	0.297	−0.079	−**0.903**
POD	−0.231	0.07	0.017	−**0.897**
PPO	−0.372	0.094	0.217	−**0.856**
Eigen variance	7.042	4.83	2.99	2.1.84
Percentage of variance	35.209	24.171	14.951	9.213
Cumulative percentage	35.209	59.380	74.331	83.544

*Eigen values are significant ≥ 0.5 which are indicated by bold letters.*

**FIGURE 3:**
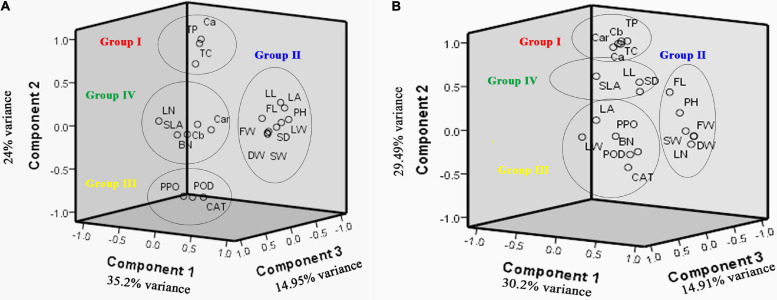
Scatter plot based on first three component analysis of 20 traits for the *A. retroflexus* **(A)** and *C. album* **(B)** populations.

A scatter plot based on the first three components explained the morphological and biochemical diversity among the measured traits (Figure 3A). Four distinct groups are determined: group I consists of total protein, Ca, and TC; group II consists of LL, LA, LW, SW, PH, FL, SD, FW, and DW; group III consists of Cat, POD, and PPO; and group IV consists of BN, LN, SLA, Cb, and Car.

#### C. album

A principal component analysis (PCA) demonstrated that the first four principal components accounted for 88.98% of the total variance (Table 4B). PC1, which explained 30.2% of the total variability, was highly correlated with PH, LL, LW, LA, SD, FW and DW, SLA, and SW. PC2 was highly correlated with Ca, Car, TP, CAT, POD, and PPO activity explaining 29.49% of the total variability. PC3 was highly correlated with the FL, BN, and LN and explained 14.91% of the total variability. PC4 was associated with Cb and TC and accounted for 14.37% of the total variability.

A scatter plot based on first three component analysis of populations demonstrated four distinct groups (Figure 3B): group I consists of total protein, Ca, Cb, TC, and Car; group II consists of FL, PH, SW, DW, FW, and LN; group III consists of LA, LW, BN, CAT, POD, and PPO; and group IV consists of SLA, LL and SD.

### Cluster Analysis

#### A. retroflexus

**FIGURE 4:**
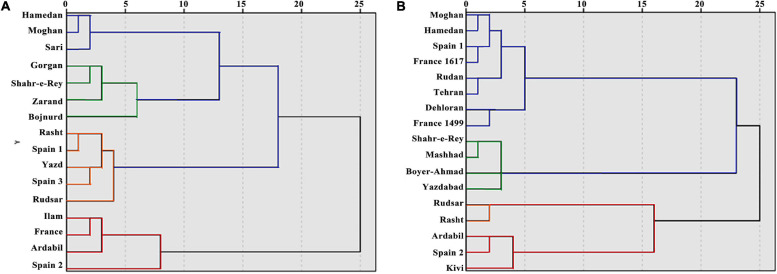
Dendrogram based on cluster analysis for 16 *A. retroflexus* **(A)** and 17 *C. album* **(B)** populations.

Cluster analysis was carried out with the Ward method, based on morphological and biochemical parameters. Generally, populations were divided into two main clusters (Figure 4A). With a decrease in the squared Euclidean distance, the populations were divided into four main sub-clusters: first sub-cluster (Hamedan, Sari, and Moghan populations), second sub-cluster (Gorgan, Shahr-e-Rey, Zarand, and Bojnurd populations), third sub-cluster (Rasht, Rudsar, Yazd, Spain 1, and Spain 3 populations), and fourth sub-cluster (Ilam, France, Ardabil, and Spain 2 populations). The results of canonical detection function analysis to determine the best cut-off point showed more differentiation with four groups (Table 5).

**TABLE 5:** Discriminant analysis to determine the cut-off point dendrogram of cluster analysis in *A. retroflexus* **(A)** and *C. album* **(B)** populations.

**Number of groups**	**Wilks’ lambda**	**Chi-square**	**Significance level**
**(A)**			
2	0.007	53.843	0.000
3	0.093	26.128	0.000
4	0.428	9.344	0.009
**(B)**			
2	0.000	138.831	0.000
3	0.004	55.988	0.000
4	0.095	23.531	0.001

#### C. album

Populations were divided into two main clusters and four sub-clusters, which was confirmed with canonical detection function analysis (Figure 4B and Table 5): first sub-cluster (Rudan, France 1617, France 1499, Tehran, Dehloran, Moghan, Hamedan, and Spain 1 populations), second sub-cluster (Boyer-Ahmad, Shahr-e-Ray, Mashhad, and Yazdabad populations), third sub-cluster (Rudsar and Rasht populations), and fourth sub-cluster (Ardabil, Kivi, and Spain 2 populations).

### Canonical Correlation Analysis

Since 99% of trait-related changes are justified by Köppen climate classification, this function was used to interpret the correlation of two sets of variables in *A. retroflexus and C. album*.

#### A. retroflexus

According to results, Cfa and Bwk climate provided relatively positive correlation with PH, FL, BN, FW, DW, SW, Ca, Car, and antioxidant enzymes and negative correlation with LL, LW, LA, LN, SD, SLA, Cb, TC, and TP. In Csa and Bsa climate, the results were the opposite of the above. The traits were not very affected by the Dsa climate (Table 6A).

**TABLE 6:** Canonical correlations between Köppen climate classification and morphological and biochemical traits in *A. retroflexus* **(A)** and *C. album* **(B)** populations.

**(A)**																				
First function correlation		0.999																		

**Climate classification**		**Cfa**				**Csa**				**Bsk**				**Bwk**			**Dsa**			

Function 1		−0.323				0.269				0.679				−0.731			0.096			

**Traits**	**PH**	**FL**	**LL**	**LW**	**LA**	**LN**	**BN**	**SD**	**FW**	**DW**	**SLA**	**SW**	**Ca**	**Cb**	**TC**	**Car**	**TP**	**CAT**	**POD**	**PPO**

Function 1	−0.115	−0.023	0.115	0.07	0.081	0.042	−0.021	0.22	−0.1	−0.08	0.256	−0.069	−0.02	0.11	0.03	−0.15	0.08	−0.093	−0.16	−0.2

**(B)**																				

First function correlation		0.999																		

**Climate classification**		**Cfa**			**Csa**			**Bsk**			**Dsa**			**Bsh**			**Bwh**			

Function 1		0.59			0.25			−0.28			0.05			−0.25			−0.8			

**Traits**	**PH**	**FL**	**LL**	**LW**	**LA**	**LN**	**BN**	**SD**	**FW**	**DW**	**SLA**	**SW**	**Ca**	**Cb**	**TC**	**Car**	**TP**	**CAT**	**POD**	**PPO**

Function 1	0.17	−0.6	0.006	0.36	0.3	0.29	0.44	−0.02	0.26	0.25	−0.54	0.32	−0.14	−0.2	−0.16	−0.33	−0.19	0.43	0.4	0.62

#### C. album

Results showed positive correlations between Bsh, Bsk, and Bwh climate and FL, SLA, SD, TP, and leaf photosynthetic pigments, moreover negative correlations with PH, LL, LW, LA, LN, BN, FW, DW, SW, and antioxidant enzymes. In Csa and Cfa climate, the results were the opposite of the above (Table 6B).

## Discussion

We set out to understand the morphological and biochemical traits of invasive weed populations for two main reasons. The first is that by characterizing these traits from populations collected from different locations, we measure the variability that is possible within and between populations and therefore quantify how variable these traits can be. Moreover, as the collection locations have different climates, we can understand better the weeds capacity to be shaped by those climatic zones. As the measured traits are under environmental as well as genetic control, we grew these populations under common garden conditions to ensure any differences we observe in the measured traits were driven by heritable differences in the populations. The second reason to study these traits is that well-characterized collections of wild populations of weeds are a useful resource for plant breeders as they provide information to guide crop improvement through gene introgression, population selection, and conventional breeding practices (Sagnard et al., 2011; Adamczyk-Chauvat et al., 2017; Neve, 2018). Since the genetic resources of weeds remain largely unexplored, understanding the extent of variability in a suite of morphological and biochemical traits will act as a primary effort to simplify improvement of cultivated plants (Andini et al., 2013).

In this study, we measured 12 morphological and eight biochemical traits of 16 *A. retroflexus* L and 17 *C. album* L. populations. Morphological traits differed significantly within the species. For instance, the BN, FW and DW, LN, LA, and SD differed among the *A. retroflexus* L. populations, and FL, LA, FW and DW, and LN were significantly different among the *C. album* L. Similarly, the measured biochemical traits also varied significantly. TP, POD activity, and Ca in *A. retroflexus* L. and TP, Car content, CAT, and POD activity in *C. album* L. all demonstrated a high coefficient of variation, therefore, high diversity among populations. These traits provide key morphological and biochemical descriptors for each of the major type of weedy population.

Principal component analysis of these data indicated that a combination of PH, LL, LW, LA, SD, FW and DW, SLA, and SW explained the most variability of *A. retroflexus*, while PH, LL, LW, LA, SD, FW and DW, SLA, and SW drove the variability of *C. album*. Scatter plot based on first three components of the PCA indicated that Group I reflected photosynthetic pigments, whereas Group III represented enzymatic activity. Group II and Group IV may indicate morphological traits among the studied *A. retroflexus* and *C. album* populations.

Canonical correlation analysis suggested that areas classified as Cfa and Bwk climates according to the Köppen climate classification system had more value of PPO, POD, and Car, and Bsk and Csa climates had more values of SD, LL, and SLA in *A. retroflexus* L. Similar analysis for *C. album* showed that Bwh, Bsk, and Bsh climates had more value of FL, SLA, and Car, while Cfa and Csa had more value of PPO, POD, and CAT. The analysis also showed that Hamedan and Moghan, Ardabil, and Spain 2 consistently cluster together in both species, but they are classified in different climate conditions. So, measured values among populations showed different results in similar climate classification from which they were collected. Therefore, the climate from which the population was collected is not a good predictor of morphology or biochemistry.

Based on the morphological and biochemical traits, cluster analysis established the phylogenetic relationship among the *A. retroflexus* and *C. album* populations. The dendrogram revealed no separate group among populations according to Köppen climate classification which supports the conclusion that there is a high level of morphological and biochemical diversity among them.

Variability observed among populations is not surprising since a high level of genetic heterogeneity is expected in plant species that are able to grow in a wide range of environmental conditions. Morphological differences have been reported in ecotypes and populations of many weeds (Bajwa et al., 2017; Van Etten et al., 2017; Le et al., 2020). A higher level of variability in morphological parameters is maintained in many of the weedy or wild relatives of crop plants (Pickersgill, 1981; Hubner et al., 2003). In fact, identification of weed species based solely on their morphological traits can be difficult (Sammour et al., 2012; Khaing et al., 2013) as weeds can exhibit a large number of morphs depending on the environment in which they are grown. The observed variation in morphological appearance might be explained in three possible ways: (1) naturally existing variations (Chan and Sun, 1997); (2) mixed mating system that may facilitate the natural introgression process; (3) polyploidy, leading to gene combination, might have resulted in higher morphological variation (Andini et al., 2013). Weedy plants are regarded as rich sources of variation and a repository of genetic diversity. These weedy populations are known to be able to survive in a large variety of habitats (Frankton and Mulligan, 1987) and the populations studied were collected from a variety of locations across their range; therefore, it is unsurprising that the different selection pressures they faced in their past have shaped the morphologies they adopt in a common garden experiment. Although self-pollination is more likely to occur, Amaranths can also cross pollinate through wind, with mean outcrossing rates ranging from 4 to 34% (Kulakow and Hauptli, 1994); therefore, Amaranths have the capacity to maintain beneficial traits as well as accumulate new ones. Polyploidy is common among plant species and recent large-scale transcriptomics indicates that whole-genome duplications have occurred repeatedly throughout flowering plants evolution (Leebens-Mack et al., 2019).

This research suggests that these heritable morphological and biochemical traits vary between populations from similar climate and suggests the local environments they have adapted to have affected the way the trait was selected. Our data are similar to other studies done with Amaranths. Andini et al. (2013) assessed the variations in morphology of Indonesian Amaranths and compared them with the worldwide variation. They proposed high levels of variability for most morphological traits. Thapa and Blair (2018) evaluated the morphological diversity of close to 300 cultivated grain Amaranths and their wild relatives from two gene banks through field assessments of leaf, flower, and grain characteristics. They concluded that the amaranth collection was a source of diversity traits and adaptation traits. Some other studies have showed that the variability of morphological traits is affected by a combination of species, climate, and soil factors (Reich et al., 2007; Han et al., 2011; Liu et al., 2012; Li et al., 2018).

In our investigation, FW showed highest significant and positive correlations with DW; moreover, TP showed highest significant and negative correlations with Ca in both species. SLA showed negative correlations with Ca which is inversely related to leaf thickness and density. At a given cellular composition, leaves of lower SLA typically have higher pigment concentrations per area due to the additional thickness of mesophyll tissue (Wright et al., 2004).

Biochemical parameters, namely, leaf photosynthetic pigments and antioxidant enzymes, were found to differ among the populations of these weed species. Weed species overcome stress more easily than cultivated plants by activating various metabolic and biochemical processes (Pavlović et al., 2014). Chlorophylls are essential for photosynthesis and their amounts can directly influence plant photosynthetic ability and biomass (Curran et al., 1990; Filella et al., 1995). Besides chlorophylls, Car are also essential for the photosynthesis process (Ong and Tee, 1992) protecting chlorophylls from photo-oxidative destruction (Giri et al., 2013). In this study, wide variations of leaf photosynthetic pigments were measured in the *A. retroflexus* and *C. album* populations. This study has identified photosynthetically efficient populations which could be used in improvement programs for cultivated grain Amaranths (Hussain and Reigosa, 2015; Zhang et al., 2016).

We also detect a significant variation in antioxidant enzyme activities among the studied various *A. retroflexus* and *C album* populations. Factors such as season, area, sampling site, water, and soil nutrients affect protein content (Sigua et al., 2012). The antioxidant enzyme activities decrease reactive oxygen species (ROS) and protect plant cells from oxidative damage under stressful conditions (Chaves and Oliveira, 2004). The disparate antioxidant potential of the *A. retroflexus* and *C. album* populations could alter their biotic and abiotic stress tolerance or resistance. According to Slabbert and Krüger (2014), greenhouse screening for leaf antioxidative enzymes production in amaranth demonstrated ecotype variation.

Our results suggest that when chlorophylls, Car, and soluble protein contents were reduced in different populations, the activities of antioxidant enzymes were increased. Even under favorable conditions, ROS production is carried out as the result of different metabolic processes and toxic oxygen derivatives are produced as a result of different stresses. Plants adopt effective systems for scavenging active oxygen species that support them against destructive oxidative reactions (Foyer et al., 1994). Antioxidant enzymes act as key elements in the defense mechanisms. Many changes have been observed in the activities of antioxidant enzymes in different ecotypes of plants (Aziz and Larher, 1998). The efficacy of the antioxidant defense system can likewise lead to high tolerance to different climate (Coelho et al., 2017).

Generally, TC concentrations showed a significant negative correlation with the level of antioxidant activities. The reaction centers of photosystem I and photosystem II are the major sites of ROS generation in the chloroplast thylakoids (Asada, 2006). One of the key factors that affect the balance between the damage and restoration of the photosynthetic activity is the relationship between the stability of the oxidative stress and the activity of the antioxidant system (Kreslavski et al., 2009). The reduced electron acceptors accumulation may increase the generation of ROS and lead to oxidative injuries. These injuries could enhance Cb degradation or the prevention of its biosynthesis, damage PSII components, and inactivate chloroplast enzymes (Cui et al., 2006). These inter-relationships among SLA, pigment concentrations, and antioxidant activities highlight the existence of a constellation of functional traits that shifts in a coordinated way during the adaptation of *A. retroflexus* L. and *C. album* L. populations to diverse environmental conditions.

## Conclusion

Populations differed significantly in studied morphological and biochemical traits. This variability is anticipated to affect the ability of specific populations to compete with other plants and response to herbicides, biotic, and abiotic stresses. Successful weed management must target the removal of biomass to limit new seed dispersal and detection strategies of new populations. Source regions may be more suited than others to cope with current and future environmental changes, although measured differences among populations are directly related to genetic differences and maternal effects. Further studies are needed to confirm these aspects for a better characterization and understanding of the strategies and abilities of invasive populations to grow and reproduce in novel environments. This understanding is essential to improve management plans particularly in the context of changing environmental conditions and providing information for propagation, domestication, and breeding programs, as well as conservation of genetic resources for plant species (Pickersgill, 1981). The existing diversity could further add new genetic information in global gene pool of weedy species. In addition, the results showed that many field traits have promise for genome analysis in the future, where combining molecular marker data with agro-morphology can identify genes for weed populations control.

## Data Availability Statement

The authors acknowledge that the data presented in this study must be deposited and made publicly available in an acceptable repository, prior to publication. Frontiers cannot accept a manuscript that does not adhere to our open data policies.

## Author Contributions

SH performed the experiments, data collection, data analysis, figure preparation, and writing of the manuscript. MA conceived the original data, formulated the research plan, oversaw the research, and writing of the manuscript. AT, MM, DW-R, DM, and TT contributed to data analysis and writing of the manuscript. All authors contributed to the article and approved the submitted version.

## Conflict of Interest

The authors declare that the research was conducted in the absence of any commercial or financial relationships that could be construed as a potential conflict of interest.
